# BACE1 is at the crossroad of a toxic vicious cycle involving cellular stress and β-amyloid production in Alzheimer’s disease

**DOI:** 10.1186/1750-1326-7-52

**Published:** 2012-10-05

**Authors:** Linda Chami, Frédéric Checler

**Affiliations:** 1 Institut de Pharmacologie Moléculaire et Cellulaire, UMR7275 CNRS/UNSA, 06560, Valbonne, France; 2 Team labelized Fondation pour la Recherche Médicale, 660 route des Lucioles, Sophia Antipolis, 06560, Valbonne, France; 3 LABEX (Laboratory of Excellence), 660 route des Lucioles, Sophia Antipolis, 06560, Valbonne, France

**Keywords:** Alzheimer’s disease, BACE1, Inflammation, Oxidative stress, Calcium

## Abstract

Alzheimer’s disease (AD) is a complex age-related pathology, the etiology of which has not been firmly delineated. Among various histological stigmata, AD-affected brains display several cellular dysfunctions reflecting enhanced oxidative stress, inflammation process and calcium homeostasis disturbance. Most of these alterations are directly or indirectly linked to amyloid β-peptides (Aβ), the production, molecular nature and biophysical properties of which likely conditions the degenerative process. It is particularly noticeable that, in a reverse control process, the above-described cellular dysfunctions alter Aβ peptides levels. β-secretase βAPP-cleaving enzyme 1 (BACE1) is a key molecular contributor of this cross-talk. This enzyme is responsible for the primary cleavage generating the N-terminus of “full length” Aβ peptides and is also transcriptionally induced by several cellular stresses. This review summarizes data linking brain insults to AD-like pathology and documents the key role of BACE1 at the cross-road of a vicious cycle contributing to Aβ production.

## The amyloid beta peptides

Alzheimer’s disease patients show progressive and irreversible memory and cognitive impairments, ultimately leading to the loss of their autonomy. This disabling disease is the first cause of dementia in the elderly population. Histopathological lesions include extracellular senile plaques mainly composed of a set of hydrophobic peptides referred to as amyloid β-peptides (A β), intracellular neurofibrillary tangles due to abnormally phosphorylated tau protein, local inflammation characterized by activated microglia and astrocytes, and neuronal loss [1]. Several risk factors such as aging, brain insults (stroke, traumatic injury), cardiovascular diseases (hypertension), or metabolic diseases (diabetes mellitus, hypercholesterolemia, obesity) [2] as well as genetic risk factors [3] have been identified but the etiology of the disease is far from being fully understood.

Aβ peptides composing the core of senile plaques are mainly produced by neuronal cells [4] and are proteolytically derived from a transmembrane precursor protein, the βamyloid precursor protein (βAPP). βAPP undergoes subsequent cleavages by β- and γ-secretases that ultimately generate Aβ peptides. An alternative and prominent processing of βAPP by α-secretase takes place in the middle of the Aβ domain of βAPP and is regarded as a physiological non-amyloidogenic pathway [5].

Even if the etiology of AD is still a matter of discussion, it is generally admitted that, if not acting as the initial trigger, Aβ peptides at least contribute to AD pathogenesis [6]. This reasonable statement is supported by genetic data. Thus, mutations responsible for early onset and aggressive AD cases affect three genes encoding proteins involved in Aβ production, namely βAPP, and presenilin 1 and 2 [7]. All these mutations modulate the endogenous levels or nature of Aβ peptides [5]. More recently, an additional genetic clue came from the observation that a novel mutation on βAPP that partly prevents its β-secretase-mediated cleavage and thereby reducing Aβ load, indeed protected bearers from AD in an Icelanders cohort [8].

Various Aβ peptides species are found in senile deposits as well as inside cells. Their nature and length can vary drastically. Genuine “full length” Aβ peptides, that are Aβ1-40 or Aβ1-42, can undergo a variety of secondary proteolytic cleavages including N-terminal truncation and cyclisation [9,10]. Moreover, monomeric soluble Aβ peptides could associate to form small soluble aggregates including oligomers and protofibrils. Soluble oligomeric species apparently display higher toxic potential for cells than Aβ monomers [11,12]. Therefore, the pathology likely results from modifications of the nature and concentration of Aβ peptides, an alteration of their biophysical properties and aggregated state, and a change in their subcellular production and accumulation that are likely underlying Aβ-associated toxicity.

In sporadic cases of AD, there is no evidence for an up-regulation of Aβ production and it is widely admitted that Aβ accumulation derives from impairment/alteration of its degradation/clearance. Amyloid peptides are mainly degraded enzymatically by neprilysin, but also and, likely to a lesser extent, by insulin degrading enzyme (IDE), endothelin-converting enzyme (ECE), angiotensin-converting enzyme (ACE), and plasmin [13]. Neprilysin mRNA and proteins are reduced in brain areas vulnerable to amyloid deposits [14] as is neprilysin activity in AD brains [15].

βAPP and its proteolytic fragments are involved in complex networks and several feedback loops have been suggested [16]. Furthermore Aβ would be able to induce its own production. Thus, the treatment of human NT2N neurons with Aβ peptide increased βAPP processing and production of Aβ peptides [17]. Aβ peptide can activate its own production by binding to the promoters of βAPP and BACE1, as Aβ has been recently shown to display transcription factor properties [18,19]. Furthermore, more related to the purpose of the present review, Aβ can also indirectly activate its production by generating various cellular dysfunctions, as detailed below.

## The β-secretase βAPP-cleaving enzyme 1

BACE1 (Asp2, memapsin 2), a single transmembrane aspartyl-protease, was identified in 1999 as the major β-secretase-like protein [20-24]. Thus, brains and primary cortical cultures derived from BACE1 knock-out mice [25-27] are devoid of β-secretase-like activity and do not produce Aβ. BACE1 is mainly expressed in neurons and in reactive astrocytes [4], in the Golgi apparatus and endosomes of cells, where amyloid peptides are mainly produced [28]. β-cleavage of βAPP is the rate-limiting step in Aβ generation [28] and therefore corresponds to an interesting therapeutic target for a strategy aimed at reducing Aβ production. BACE1 is not fully selective for βAPP and other substrates have been identified, suggesting an additional role of BACE1 in immunity or sodium channels function [29]. BACE1 knockout mice are viable and fertile [25] but recent data indicate that these mice could harbor axon hypomyelination [30,31], schizophrenia-like [32] and epileptic-like [33] behaviors.

Environmental [34,35] and cellular [36] stresses induce the expression of BACE1. BACE1 promoter harbors functional binding sites for numerous transcription factors including specificity protein 1 (Sp1; [37]), Yin Yang 1 (YY1; [38]), the peroxisome proliferator-activated receptor γ (PPARγ [39]), the nuclear factor-κB (NF-κB; [40,41]), the hypoxia-inducible factor 1(HIF-1; [42]), and signal transducer and activator of transcription 3 (STAT3; [43]). BACE1 activity increases with age [44] and pathology. In AD brains, BACE1 is elevated in regions that develop amyloid plaques and more particularly, in neurons surrounding amyloid plaques [41,45,46]. The purpose of this review is to describe transcriptional regulations of BACE1. BACE1 regulation by translational modification, maturation and trafficking will not be treated as they have been nicely reviewed elsewhere [29,47-49].

As stated above, BACE1 is a stress-induced protease. Oxidative stress, inflammation, calcium homeostasis disturbance, hypoxia, ischemia and trauma conditions that occur in AD activate BACE1 (see below). The activation of BACE1 due to transcriptional deregulation could contribute and possibly accelerate AD pathology by increasing Aβ production. As Aβ42 peptide can activate BACE1, [50-53], a positive regulatory loop setting a vicious cycle can be delineated and is described in details below.

## Oxidative stress

### Oxidative stress in AD

Reactive oxygen species (ROS) and reactive nitrogen species are normal products of cell metabolism. Their concentration is balanced by antioxidant factors and is associated to either beneficial or deleterious effects. Low to moderate free radicals concentrations are part of the physiological cellular signaling system and defense mechanisms against infection agents. Conversely, excessive oxidant conditions trigger oxidative stress that turns out to be toxic for cells by damaging lipids, proteins or nucleic acids, ultimately leading to cell death [54]. Oxidative damage further impairs the antioxidant defense and maintains oxidative burden in the cells [2,54].

Lifespan accumulation of free radicals results in age-associated oxidative stress, the damages of which cause cellular and organism senescence [2,55]. Oxidative stress is associated to AD as an early event [56-58]. Oxidative stress contributes to AD; various mechanisms have been identified [59], such as the oxidative inactivation of the peptidyl-prolyl *cis/trans* isomerase 1 (Pin1) that affects its regulation of βAPP production and tau dephosphorylation [60]. Interestingly, amyloid deposits and neurofibrillary tangles have been postulated to be part of antioxidant strategies developed by the organism in response to age-related increase in oxidative stress (reviewed by [54,61]).

### Aβ generates oxidative stress

Aβ peptides trigger oxidative stress *in vitro* and *in vivo* (reviewed by [59]). On the one hand, Aβ induces ROS generation, with a possible contribution of metal ions. Copper and iron are present in amyloid deposits and their reduction by Aβ produces ROS. The more powerful are the Aβ species considered as the more toxic. Thus, Aβ1-42 had greater iron and copper reduction potential than Aβ1-40 *in vitro*[62,63], and prefibrillar and oligomeric forms of Aβ1-42 induced higher oxidative stress than fibrillar Aβ1-42 in neuronal cells [50]. On the other hand, Aβ peptides contribute to oxidative stress by impairing the cellular antioxidant systems. Thus continuous ventricular Aβ infusion reduced the immunoreactivity of the Mn-superoxide dismutase (Mn-SOD) and proteins of the glutathione antioxidant system in rats [64].

### Oxidative stress activates BACE1

BACE1 activity is positively correlated to oxidative stress markers in AD brains [65]. Treatment of cells with various oxidants increases BACE1 transcription, expression and activity [66,67]. Oxidative stress regulates the γ-secretase activity as well [51], and treated cells produce more Aβ peptides [68,69].

The JNK pathway is activated in response to oxidative stress, inflammatory cytokines and excitotoxic stimuli; then activated JNK positively regulates inflammation and apoptosis [70]. JNK is activated by Aβ in neuronal cultures [71,72] and high levels of activated JNK have been reported in degenerating neurons of human AD brains [73] or transgenic mice [72]. JNK pathway also contributes to Aβ toxicity *in vitro*[71,74] and production. Thus JNK gene manipulation or pharmacological blockade prevented oxidative stress-induced upregulation of BACE1 in mouse fibroblasts as well as in mice [51]. Therefore, the c-Jun N-terminal kinases (JNK) pathway is involved in BACE1 regulation by oxidative stress.

### Aβ peptides regulate BACE1 by generating oxidative stress

**Figure 1:**
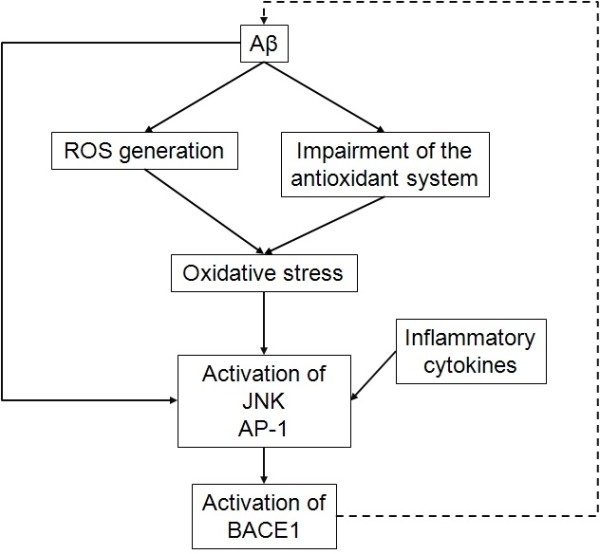
**Oxidative stress mediates Aβ-induced BACE1 transcriptional activation.** Aβ peptides trigger oxidative stress by inducing ROS generation and impairing the antioxidant system. Oxidative stress and inflammatory cytokines activates JNK, then its transcription factor AP-1 upregulates BACE1. As BACE1 produces Aβ peptides, a vicious cycle is established. *Aβ, amyloid peptide; AP-1, activator protein-1; BACE1, β-secretase βAPP cleaving enzyme 1; JNK, c-Jun N-terminal kinases; ROS, reactive oxygen species*.

As detailed before, Aβ induces oxidative stress and the latter activates BACE1. Hence Aβ indirectly regulates BACE1 by generating oxidative stress. The JNK pathway and its major transcription factor activator protein-1 (AP-1) are involved in this regulation. Guglielmotto and collaborators demonstrated that pharmacological inhibition and gene depletion or mutation of JNK or downstream proteins abolished Aβ42 control of BACE1 activation in murine fibroblasts [72]. Therefore, by inducing oxidative stress and activation of BACE1, Aβ regulates its own production (Figure 1).

## Inflammation

### Inflammation in AD

In response to an injurious stimulus, the organism settles inflammation until the physiological homeostasis is restored. In the central nervous system, microglia is the major actor of inflammation. Resting glial cells become motile when activated and surrounds damaged cells, clear-off cellular debris and release inflammatory agents such as cytokines, chemokines, complement factors, and free radical species [75]. These signals activate astrocytes that undergo morphological and functional changes, and thus participate to the inflammatory process [75]. Neurons contribute to microglial activation by production of pro-inflammatory cytokines and complement proteins [76].

Neuroinflammation accompanies normal aging. Aging rodents harbor increased activated microglia and astrocytes together with an increase of pro-inflammatory cytokines or a decrease of anti-inflammatory cytokines [77]. Local and chronic neuroinflammation is a constant feature of AD, and is characterized by activated microglia and astrocytes surrounding amyloid plaques and neurofibrillary tangles [78]. Accordingly, elevated levels of cytokines are measured in AD brains [79]. Inflammation can exert both neuroprotective and neurotoxic functions that are directly linked to the duration of the inflammatory process. Acute inflammation is considered to be beneficial by contributing to restore the physiological integrity of tissues. Activated glial cells are thus beneficial since they clear Aβ by phagocytosis and degradation [80,81]. On the other hand, sustained inflammation observed in AD brains, probably in response to continuous accumulation of Aβ peptides and cellular debris, can be toxic to neurons since inflammatory mediators such as ROS, cytokines and chemokines could directly take part to neurite retraction, neuronal dysfunction and neuronal death [80,82]. The metabolites released by activated microglia add to the dual effect of inflammation, as they can be neurotoxic, antioxidant, pro- or anti-inflammatory. The role of inflammation in AD is therefore the resultant of various cellular and molecular events.

### Aβ peptides are pro-inflammatory

Aβ treatment induces an activation of microglial and astrocytic cells, leading to the release of inflammatory factors [75,83,84]. Aβ activate glial cells by direct binding to microglial cell surface receptors [85], such as the receptor for advanced glycation end products (RAGE, [86]), by direct activation of the complement system [87], or by generating oxidative stress [88].

The transcription factor NF-κB is activated in response to various stresses [88]. NF-κB is induced by inflammation- and oxidative stress-linked conditions such as release of cytokines [88] and ROS [89], as well as ischemia [90] or traumatic brain injury [91] in rats. NF-κB has a dual role in inflammation, since it is associated to pro-inflammatory or anti-inflammatory genes induction during the onset or the management of inflammation, respectively [92].

Aβ peptides activate NF-κB in neurons and astrocytes [17,93,94]. The lowest Aβ concentrations were the more efficient to activate NF-κB [93,94]. NF-κB activation has been reported in human cortex areas affected by the pathology, particularly in cells surrounding senile plaques [41,93-95]. The role of NF-κB activation remains unclear. Several works suggested a protective cellular response to Aβ-induced cell death [94,96]. However other studies indicated that NF-κB could contribute to Aβ-associated toxicity, as inhibition of NF-κB reduced Aβ-induced neuronal death [17,97].

### Inflammation activates BACE1

The well-known inflammation inducer lipopolysaccharide (LPS) increases βAPP expression and processing in Swedish-βAPP transgenic mice [98]. LPS and inflammation activate the transcription factor NF-κB, for which BACE1 promoter harbors a highly conserved binding site [99] that is functional [40,41]. NF-κB physiologically represses BACE1 transcription *in vitro*[40,100], therefore limiting Aβ production.

However inflammatory conditions could favor Aβ production by switching the NF-κB inhibition of BACE1 transcription towards an activation process as suggested by many studies. Thus NF-κB activates BACE1 promoter, expression and enzymatic activity in activated astrocytes and Aβ-exposed or Aβ-overproducing cells, leading to increased Aβ production [40,41,52,100]. *In vivo*, the modulation of NF-κB activity by non-steroidal anti-inflammatory drugs [101], natural compounds [102,103] or by targeting upstream receptors of the NF-κB activation pathway [58,104], all affect Aβ production. In transgenic mice NF-κB activates βAPP levels [103], BACE1 promoter activity [104], expression [102,105] and enzymatic activity [102,103] as well as γ-secretase activity [103] and Aβ production [101-103].

NF-κB-dependent regulation of BACE1 is therefore ambivalent, since NF-κB would physiologically repress BACE1 transcription, but would convert into an activator of BACE1 in cells exposed to an Aβ overload [40,100]. This could be explained by the activation of different NF-κB heterodimers yielded in a stimulus-dependent manner [40] even if this remains to be definitely established.

Other mediators of inflammation contribute to the regulation of BACE1. PPARγ are nuclear receptors that inhibit pro-inflammatory gene expression such as NF-κB-regulated genes, and are targeted by some nonsteroidal anti-inflammatory drugs (NSAID, [106]). PPARγ inhibits BACE1 transcription through a functional PPAR response element on BACE1 promoter and interferes with the cytokines-induced Aβ production, as demonstrated in cells and confirmed in transgenic mice and human brains [39,107,108]. PPARγ agonists have additional beneficial effects on Aβ peptides production by increasing βAPP or BACE1 degradation [109,110].

Prolonged inflammation could favor Aβ production by activating astrocytes, as demonstrated by various *in vitro* and *in vivo* studies. Thus, chronic stress, pro-inflammatory cytokines or Aβ42 itself increase BACE1 levels and activity as well as βAPP levels in astrocytes. The transcription factors NF-κB, YY1 and STAT1 could account for the stress-induced increase of BACE1 transcription in astrocytes [38,40,111-115] that are observed in the vicinity of amyloid plaques in both aged Tg2576 mice and AD-affected brains [116]. However a recent study challenges these results by showing a reduced Aβ secretion in response to cytokine stimulation of cultured rat astrocytes, in which the β-secretase activity would be accounted for by the BACE1 homolog, BACE2 [117].

### Aβ peptides regulate their own production by triggering NF-κB-mediated BACE1 activation

**Figure 2:**
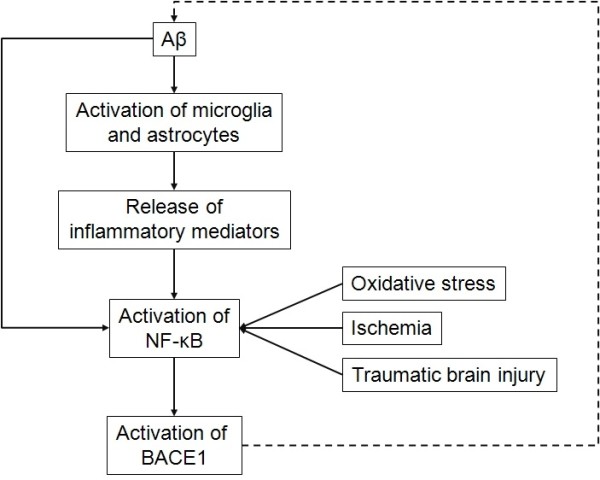
**Inflammation mediates Aβ-induced BACE1 transcriptional activation.** Aβ peptides are pro-inflammatory. They activate microglia and astrocytes that release inflammatory mediators. Those activate NF-κB, which is also activated by oxidative stress, ischemia or traumatic brain injury. Pathological activation of NF-κB activates BACE1 transcription, thus increasing Aβ peptides levels and feeding a vicious cycle. *Aβ, amyloid peptide; BACE1, β-secretase βAPP cleaving enzyme 1; NF-κB, nuclear factor-κB*.

At supraphysiological levels, Aβ induces an upregulation of BACE1 transcriptional activity, protein expression, enzymatic activity, and consequently intracellular accumulation and secretion of Aβ, by activating NF-κB [17,40,52,100]. BACE1 transcription is therefore activated by Aβ and by inflammation. In turn, BACE1 can promote inflammation by the production of two pro-inflammatory agents that are Aβ and the prostaglandin E2, produced by BACE1 cleavage of the membrane-bound prostaglandin E2 synthase-2 [118]. Therefore by inducing inflammation and NF-κB activation, Aβ could act on its own production (Figure 2).

## Calcium homeostasis perturbation

### Calcium signaling perturbation in AD

Calcium is a major signaling molecule involved in a variety of neuronal functions, such as neurotransmission, synaptic plasticity, excitotoxicity or apoptosis [119,120]. Aging affects calcium sensitivity and homeostasis, thereby triggering neuronal vulnerability and cell death. Oxidative stress is tightly associated to these calcium homeostasis alterations [121,122].

The calcium signaling pathway is altered in AD. Intracellular levels of calcium are increased by a disturbed entry of external calcium, an exacerbated release from the internal storage organelles endoplasmic reticulum and mitochondria, and/or an hypersensitivity of the system [121,123]. The disturbed calcium signaling alters long-term potentiation and long-term depression, thus affecting learning and memory. Finally, an overload of calcium can induce the mitochondria to trigger apoptosis and neurodegeneration [121]. The polymorphism of a calcium channel was formerly associated to an increased risk of AD. The calcium homeostasis modulator 1 (CALHM1) channel controls intracellular calcium levels and calcium-dependent α-secretase-mediated processing of βAPP [124]. A polymorphism in its gene impairs its physiological functions and favors Aβ overload. Currently, the CALHM1 polymorphism is rather considered as a genetic modifier of age at onset in AD [125].

### Aβ affects cellular calcium homeostasis

Calcium homeostasis disturbance is part of Aβ neurotoxicity (for reviews see [120-122]). Amyloid peptides increase the level of cytoplasmic calcium through several mechanisms, as suggested by the *in vitro* experiments described below. Aβ can trigger an extracellular calcium influx by stimulating membrane ion channels or receptors, such as ionotropic glutamate receptors [126]. Aβ could impair the intracellular distribution of calcium by perforating and permeabilizing the membrane to calcium via oxidative stress [120,127,128]. Noteworthy, some presenilin mutations responsible for familial AD and yielding enhanced Aβ levels, impair calcium homeostasis by deregulating internal calcium channels ryanodine receptor [129], inositol 1,4,5-triphosphate (IP3) channel [130] or sarco endoplasmic reticulum calcium ATPase (SERCA; [131]). This agreed well with our recent work showing that the overexpression of both wild-type and Swedish-mutated βAPP increased Ryanodine receptors (RyR) expression and enhanced RyR-mediated ER Ca2+ release in neuroblastoma cells as well as in transgenic mice [132]. Altering presenilin functions has an impact on calcium homeostasis by an additional mechanism. Concomitant to the generation of Aβ, the γ-secretase complex releases the βAPP intracellular domain (AICD) which acts as a transcription factor [133] involved in the transactivation of genes related to AD [134,135]. Similarly, AICD is involved in calcium signaling [136] or homeostasis in different cell culture models [137].

### Calcium disturbance activates BACE1

Calcium dysregulation promotes tau phosphorylation and Aβ accumulation in neuronal cells [138-140]. Calpain is an intracellular cystein protease regulated by calcium and abnormally activated in AD brains [141,142]. In transgenic mice brains, calpain over-activation induces amyloid deposits, tau phosphorylation, activation of astrocytes, synapse loss and cognitive impairment [141,143]. Furthermore, βAPP processing is affected as βAPP C-terminal fragments are decreased following calpain inhibition in these mice [143].

BACE1 upregulation could be mediated by cyclin-dependent kinase 5 (cdk5), which is regulated by calpain [144]. Cdk5 activates BACE1 promoter by binding of its target STAT3, therefore increasing BACE1 activity, Aβ1-40 and Aβ1-42 production in transgenic mice [43]. Another calcium-dependent transcription factor regulates BACE1 transcription. The calcium-activated nuclear factor of activated T-cells 1 (NFAT1), which is abnormally activated in transgenic mice brain [145], translocates to the nucleus, binds to BACE1 promoter, activates its transcription and increases Aβ generation, as demonstrated *in vitro*[145].

Many evidences thus imply a calcium-dependent activation of BACE1. However, two *in vitro* studies suggest that the regulation of Aβ production by calcium would be more complex. Hayley and collaborators who demonstrated a physical interaction between calcium and BACE1 reported on an activation of BACE1 activity at low calcium concentration, and conversely, a progressive reduction of BACE1 activity when increasing calcium concentration [146]. Similar results were obtained on Aβ production using thapsigargin, a pharmacological raiser of cytoplasmic calcium levels [147].

### Aβ peptides regulate BACE1 via calcium-dependent pathways

**Figure 3:**
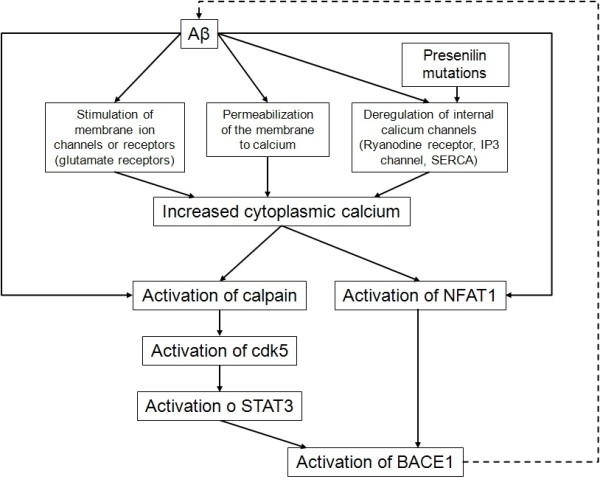
**Disturbed calcium homeostasis mediates Aβ-induced BACE1 transcriptional activation.** Aβ peptides increase cytoplasmic calcium by at least three mechanisms: stimulation of membrane ion channels or receptors; permeabilization of the membrane; and deregulation of internal calcium channels. Presenilins mutations contribute to the latter. Increased calcium then activates the calpain/cdk5/STAT3 pathway and NFAT1. The transcription factors STAT3 and NFAT1 upregulate BACE1, which then produces more Aβ peptides and a positive feedback mechanism is set up. *Aβ, amyloid peptide; BACE1, β-secretase βAPP cleaving enzyme 1; cdk5, cyclin-dependent kinase 5; IP3, inositol 1,4,5-triphosphate; NFAT1, nuclear factor of activated T-cells 1; SERCA, sarco endoplasmic reticulum calcium ATPase; STAT, signal transducer and activator of transcription*.

As detailed above, impaired calcium homeostasis activates BACE1 via activation of NFAT1 and the calpain/cdk5/STAT3 pathway. By altering calcium signaling, Aβ regulates BACE1 through both pathways. Aβ treatment of cultured neurons activated calpain, cdk5, NFAT1 [145,148] and increased BACE1 expression [143,145] that was reduced by calpain inhibition [143] or calcineurin-mediated NFAT1 inhibition [145]. Therefore calcium is another intermediate by which Aβ upregulates BACE1, and thus its own production (Figure 3).

## Advanced glycation end (AGE) products

### AGEs in AD

AGEs are normal products of cellular metabolism. They result from irreversible post-translational modifications of proteins on which monosaccharides are grafted by non-enzymatic mechanisms. By generating protease-resistant peptides and proteins, this reaction leads to protein deposition and amyloidosis [149]. AGEs accumulate in aged tissue and contribute to the age-related deterioration of cellular functions [150]. AGE production can be enhanced in pathological contexts such as diabetes mellitus-associated hyperglycemia, inflammation, and hypoxia [149,151]. AGEs pathogenicity is linked to the concomitant oxidative stress generated during their formation, to their interaction with its receptor RAGE [152], or by the accumulation of non-degradable proteins [149,151]. Furthermore, AGEs binding to RAGE intensifies inflammation by activation of NF-κB and by release of pro-inflammatory cytokines [153,154]. In turn, NF-κB transactivates RAGE promoter [155]. Finally, AGEs compete with other physiological ligands interacting with RAGE, such as growth or differentiation factors [149,151].

Cerebral levels of AGEs are increased in human AD brains, especially in neurofibrillary tangles and amyloid deposits [156-159]. Tau and Aβ peptides are indeed substrates for glycation, which contributes to their pathogenicity. Thus *in vitro* studies showed that tau glycation impairs its ability to bind to tubulin [160], and AGEs favor Aβ peptides aggregation [157,161].

### Aβ modulates the AGE/RAGE signaling cascade

Aβ peptides that can be considered as AGEs, bind to RAGE [86] and upregulate this receptor through the cytokine macrophage colony-stimulating factor (M-CSF). This amplifies RAGE sensitivity for Aβ stimulation and probably subsequent pro-inflammatory conditions settled by the microglia [162]. Arancio and collaborators highlighted the contribution of RAGE to AD phenotype. Transgenic mice overexpressing mutant βAPP and RAGE exhibited earlier cognitive abnormalities and altered synaptic function, along with an increase in NF-κB activation and amyloid deposits-associated reactive microglia and astrocytes [163].

### AGEs and RAGE activate BACE1

**Figure 4:**
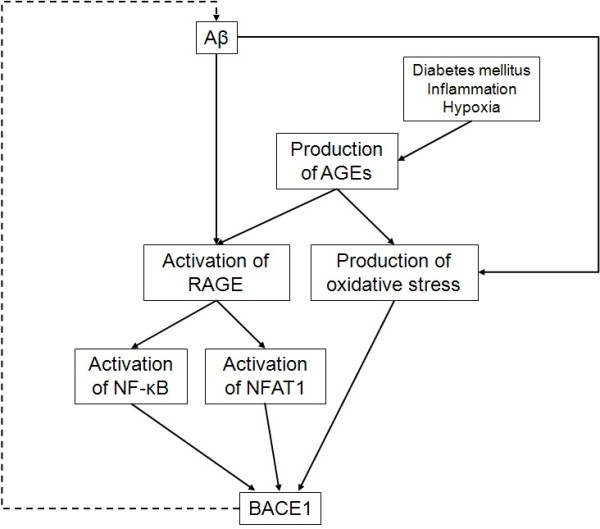
**Aβ and AGEs activate BACE1 transcription.** Aβ peptides activate RAGE. This receptor is also activated by AGEs produced during diabetes mellitus, inflammation or hypoxia. RAGE activation upregulates BACE1 by the activation of the two transcription factors NF-κB and NFAT1. Additionally, AGEs can activate BACE1 by generating oxidative stress. BACE1 contribution to Aβ peptides production then amplifies RAGE activation. *Aβ, amyloid peptide; AGE, advanced glycation end products; BACE1, β-secretase βAPP cleaving enzyme 1; NFAT1, nuclear factor of activated T-cells 1; NF-κB, nuclear factor-κB; RAGE, receptor for advanced glycation end products*.

AGEs can influence Aβ generation. AGEs induce βAPP expression by generating oxidative stress in SH-SY5Y cells [164] and in transgenic mice model of AD, RAGE injection increases Aβ accumulation and senile plaques [165]. As mentioned in this review, pro-oxidant conditions regulate BACE1. Similarly, BACE1 expression and activity are increased by the activation of RAGE in transgenic mice and SH-SY5Y cells [165]. NFAT1 could be involved in this regulation, since AGEs- or Aβ-mediated stimulation of RAGE increased cytosolic calcium concentration, NFAT1 activation and binding to BACE1 promoter in SH-SY5Y cells [165]. The NF-κB pathway seems also involved in RAGE-dependent regulation of BACE1. Thus pentosidine and glyceraldehydes-derived pyridinium, two AGEs that are increased in AD patients brains, upregulate BACE1 expression by binding with RAGE and subsequent activation of NF-κB *in vitro* and *in vivo*[166]. Therefore, RAGE activation by AGEs or Aβ activate BACE1 transcription and thereby, increases Aβ production (Figure 4).

## Brain insults

### Traumatic brain injury

Traumatic brain injury is a risk factor for AD [167]. *Post mortem* analysis of patients who had traumatic brain injury revealed deposition of Aβ peptides in brain and abnormal distribution in the cerebrospinal fluid [167,168]. This was confirmed in transgenic mice model of AD, where repetitive traumatic brain injury triggered Aβ accumulation [169]. Traumatic brain injury is followed by an increase of BACE1 mRNA, protein and activity, as well as an accumulation of βAPP and presenilin 1 [170-172].

**Figure 5:**
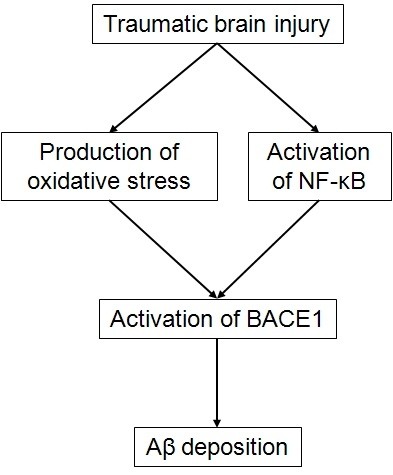
**Traumatic brain injury contributes to Aβ deposition by activating BACE1 transcription.** Traumatic brain injury activates BACE1 by inducing oxidative stress and by activating the NF-κB transcription factor. This leads to Aβ deposition. *Aβ, amyloid peptide; AGE, advanced glycation end products; BACE1, β-secretase βAPP cleaving enzyme 1; NF-κB, nuclear factor-κB*.

BACE1 activation could be due to oxidative stress and NF-κB activation following traumatic brain injury [91,169,173], as we previously described that both can upregulate BACE1 (Figure 5). In addition, BACE1 upregulation may result from an impaired degradation. The GGA (Golgi-localizing, γ-adaptin ear homology domain, ARF-binding) proteins regulate BACE1 trafficking between endosomes and Golgi apparatus [174-176]. Following head injury, activated caspases cleave GGA1 and GGA3, thereby stabilizing BACE1 [177].

BACE1 deletion attenuates brain damages due to traumatic injury. Thus learning impairment and tissue damage are attenuated in BACE1 *null* mice. BACE1 would contribute to the continuing neuronal damage after the initial injury, where apoptotic and inflammatory pathways are activated [172].

### Hypoxia

Vascular risk factors, like heart disease or stroke leading to hypoperfusion are risk factors for AD [178,179]. Hypoperfusion, that is a transient or permanent reduction in cerebral blood flow leading to subsequent hypoxia, causes a decrease in the important source of energy ATP, a perturbation of ionic gradients, an increase in cytoplasmic calcium concentration, an excitotoxic excess of extracellular glutamate, oxidative stress, and activation of pro-inflammatory pathways, ultimately leading to cell death [180].

**Figure 6:**
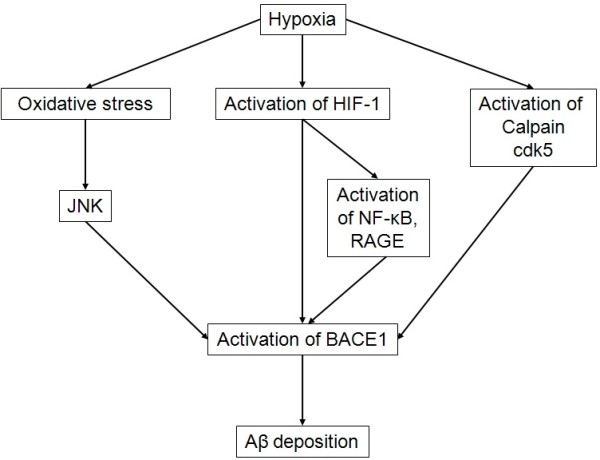
**Hypoxia contributes to Aβ deposition by activating BACE1 transcription.** Hypoxia activates BACE1 by three distinct mechanisms: generation of oxidative stress and the subsequent activation of the JNK pathway; activation of HIF-1 transcription factor which activates BACE1 promoter directly or indirectly through the activation of NF-κB and RAGE; activation of calpain and cdk5 resulting from increased calcium concentrations. By activating BACE1 transcription, hypoxia thus leads to Aβ deposition. *Aβ, amyloid peptide; AGE, advanced glycation end products; BACE1, β-secretase βAPP cleaving enzyme 1; cdk5, cyclin-dependent kinase 5; HIF-1, hypoxia-inducible factor 1; JNK, c-Jun N-terminal kinases; NF-κB, nuclear factor-κB; RAGE, receptor for advanced glycation end products*.

In response to hypoxia, BACE1 levels, maturation and activity, as well as Aβ deposition and memory deficits are increased in Swedish mutant APP mice. In this pathological condition, BACE1 transcription is activated by hypoxia-inducible factor (HIF-1), a major transcription factor induced by oxygen reduction [42,181]. Guglielmotto and collaborators [182] proposed a biphasic activation of BACE1 by hypoxia. The early phase would be characterized by the release of ROS from mitochondria and by the activation of the JNK pathway, whereas during the late phase, the HIF1α transcription factor would take over BACE1 activation. Besides oxidative stress [182], other hypoxia-linked mechanisms could contribute to BACE1 activation, such as the activation of calpain and cdk5 [183-185], or the upregulation of RAGE or NF-κB by an HIF-1α-dependent transcriptional activation [186-189]. The three mechanisms explaining hypoxia-induced BACE1 upregulation are summarized in Figure 6.

Finally, two additional post-transcriptional mechanisms contribute to elevate BACE1 levels: the phosphorylation of eIF2α subsequent to energy deprivation that translationally activates BACE1 [190]; reduction of GGA3 levels following ischemia, leading to BACE1 stabilization and increased β-secretase activity [191].

## Aβ-linked apoptosis in AD

Aβ toxicity mediated by oxidative stress, inflammation, disturbed calcium homeostasis and cellular disorder described above, leads to apoptosis. Aβ can activate the extrinsic or the intrinsic apoptotic pathways according to its aggregation state (reviewed in [11]). Aβ can directly induce apoptosis by activating the transcription of the tumor suppressor p53 [192], the expression of which is increased in AD brains [192,193]. Furthermore, by activating p53, Aβ and AICD can regulate their own production [192,194-196], since p53 has been shown to regulate some of the γ-secretase complex proteins that are presenilin 1, presenilin 2 and presenilin enhancer 2 (Pen-2) [195,197,198].

## Conclusion

Changes observed in AD brains are not necessarily causes of the disease, and could be consequences of the pathological process [199]. Most of cellular responses and adaptative processes described in this review as well as Aβ peptides can exert both protective and toxic functions according to the cellular context. For Aβ peptides, those include modulating ion channel function [200], neuronal viability [201,202], protection from glutamate and N-methyl-D-aspartic acid excitoxicities [202,203], and reduction of oxidative damage [204-206]. Aβ excess is considered to have a causative role in AD pathogenesis, but could be a protective mechanism in response to various stresses [9,204,207,208].

**Figure 7:**
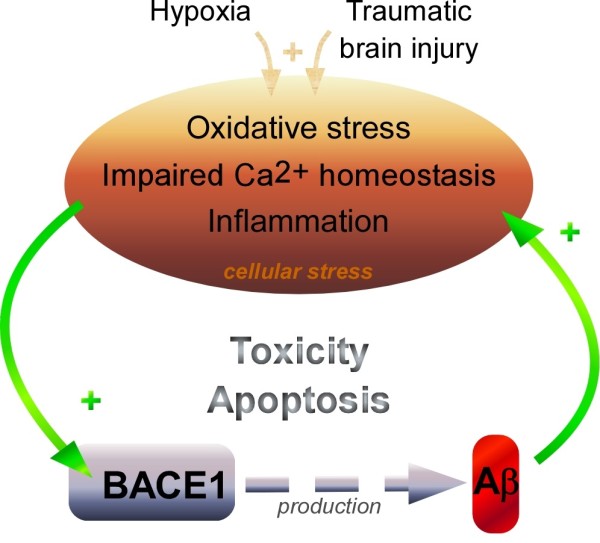
**Cellular stress, BACE1 and Aβ production are involved in a toxic vicious cycle in AD.** Various cellular dysfunctions including oxidative stress, inflammation and calcium homeostasis disturbance occur in AD-affected brains. These alterations activate the transcription of the stress-induced β-secretase BACE1 that contributes to Aβ production. Once yielded at supra-physiological levels, Aβ induces cellular stresses that, in turn activate BACE1, therefore setting up a vicious cycle. Such self-maintained toxicity can lead to cellular cell death. Brain insults like hypoxia and traumatic brain injury contribute to this scheme by inducing cellular stress.

Nevertheless*,* AD brain cells undergo various stresses mainly caused by oxidative stress, inflammation and calcium homeostasis impairment. Chronic exposition of cells to these age-related perturbations or brain insults maintains supraphysiological BACE1 levels, leading to an increased production of amyloid peptides, particularly significant since their degradation is reduced in AD. Since these peptides in turn contribute to oxidative, inflammatory and disturbed calcium conditions, this overall contributes to feed a morbid vicious cycle described in the Figure 7. According to this scheme, BACE1 activation and accompanying increase in Aβ production play a key role in the amplification of cellular dysfunctions. It should be noted that an interesting recent paper indicates that BACE1 upregulation may contribute to AD pathogenesis by disturbing synaptic functions, independently of its catalytic role in Aβ production. Thus, Chen and collaborators showed that BACE1 negatively controls the cAMP/PKA/CREB pathway by interacting adenylate cyclase. This regulation was not affected in cells devoid of Aβ. The CREB pathway is important for memory functions, and upregulation of BACE1 in mice did affect their learning and memory abilities, in the absence of βAPP fragments [209].

Since BACE1 contributes to AD pathogenesis and is essential to the cycle described in Figure 7, limiting its activity is an interesting therapeutic strategy. Inhibitors of BACE1 have been developed and improved recently. Some non-peptidic orally available compounds with good pharmacological properties reduced brain Aβ levels in AD transgenic mice and already are under phase I clinical studies. One of them successfully passed phase I trial and reduced plasma Aβ levels in AD patients (for review, see [210]). However BACE1 inhibition should not be complete to prevent potential side effects (hypomyelination, schizophrenia- and epileptic-like behaviors, hippocampal neurodegeneration [30-33,210]) linked to BACE1-associated proteolysis of other substrates. Different therapeutic strategies aimed at reducing inflammation or oxidative damage in AD did not prove to be successfull so far [211,212]. It is likely that AD treatment may need to target simultaneously distinct components/pathways to be efficient, and should be used in the early phase of development of the pathology in order to prevent irreversible damages in AD brains [211].

## Abbreviations

Aβ: amyloid peptide; ACE: angiotensin-converting enzyme; AD: Alzheimer’s disease; AGE: advanced glycation end products; AICD: βAPP intractellular domain; AP-1: activator protein-1; BACE1: β-secretase βAPP cleaving enzyme 1; βAPP: β-amyloid precursor protein; CALHM1: calcium homeostasis modulator 1; cdk5: cyclin-dependent kinase 5; ECE: endothelin-convertin enzyme; GGA: golgi-localizing γ-adaptin ear homology domain, ARF-binding; HIF-1: hypoxia-inducible factor 1; IDE: insulin degrading enzyme; *IP3*: *inositol 1,4,5-triphosphate*; JNK: c-Jun N-terminal kinases; LPS: lipopolysaccharide; M-CSF: macrophage colony-stimulating factor; NFAT1: nuclear factor of activated T-cells 1; NF-κB: nuclear factor-κB; NSAID: nonsteroidal anti-inflammatory drugs; Pen-2: presenilin enhancer 2; Pin-1: peptidyl-prolyl *cis/trans* isomerase 1; PPARγ: peroxisome proliferator-activated receptor γ; RAGE: receptor for advanced glycation end products; ROS: reactive oxygen species; SERCA: sarco endoplasmic reticulum calcium ATPase; Sp1: specificity protein 1; STAT: signal transducer and activator of transcription; YY1: Yin Yang 1.

## Competing interests

The authors declare no competing interests.

## Authors’ contributions

Manuscript drafted and edited by LC and FC. All authors read and approved the final manuscript.
